# 3,9-Dimethyl-3,9-bis­(4-nitro­phen­yl)-2,4,8,10-tetra­oxaspiro­[5.5]undeca­ne

**DOI:** 10.1107/S1600536811015017

**Published:** 2011-05-07

**Authors:** Xiaoqiang Sun, Bin Yu, Xiuqin Zhang, Xuqiang Chao, Qiang Chen

**Affiliations:** a Key Laboratory of Fine Petrochemical Engineering, Changzhou University, Changzhou 213164, People’s Republic of China; b High Technology Research Institute of Nanjing University, Changzhou 213162, People’s Republic of China

## Abstract

In the title compound, C_21_H_22_N_2_O_8_, both of the nonplanar six-membered heterocycles adopt chair conformations. The dihedral angle between the terminal benzene rings is 58.22 (11)°. Weak inter­molecular C—H⋯O inter­actions are observed in the crystal structure.

## Related literature

For general background to spiranes, see: Cismaş *et al.* (2005 ▶); Mihiş *et al.* (2008 ▶); Sun *et al.* (2010 ▶).
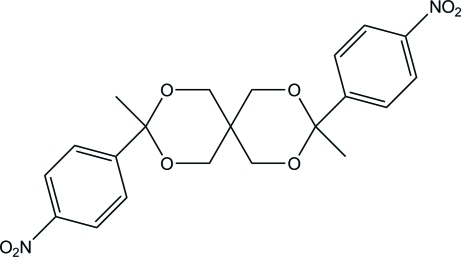


## Experimental

### 

#### Crystal data

C_21_H_22_N_2_O_8_
*M* *_r_* = 430.41
Triclinic, 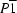

*a* = 7.4215 (12) Å
*b* = 11.8790 (18) Å
*c* = 13.522 (3) Å
α = 115.280 (4)°
β = 94.426 (4)°
γ = 103.444 (3)°
*V* = 1027.0 (3) Å^3^
*Z* = 2
Mo *K*α radiation
μ = 0.11 mm^−1^
*T* = 295 K
0.21 × 0.21 × 0.16 mm

#### Data collection

Bruker APEXII CCD diffractometer
Absorption correction: multi-scan (*SADABS*; Sheldrick, 2003 ▶) *T*_min_ = 0.976, *T*_max_ = 0.986
5588 measured reflections
3563 independent reflections
2980 reflections with *I* > 2σ(*I*)
*R*_int_ = 0.022

#### Refinement

*R*[*F* ^2^ > 2σ(*F* ^2^)] = 0.051
*wR*(*F* ^2^) = 0.187
*S* = 1.07
3563 reflections
282 parameters
12 restraints
H-atom parameters constrained
Δρ_max_ = 0.37 e Å^−3^
Δρ_min_ = −0.26 e Å^−3^

### 

Data collection: *APEX2* (Bruker, 2007 ▶); cell refinement: *SAINT* (Bruker, 2007 ▶); data reduction: *SAINT*; program(s) used to solve structure: *SHELXS97* (Sheldrick, 2008 ▶); program(s) used to refine structure: *SHELXL97* (Sheldrick, 2008 ▶); molecular graphics: *SHELXTL* (Sheldrick, 2008 ▶); software used to prepare material for publication: *SHELXTL*.

## Supplementary Material

Crystal structure: contains datablocks I, global. DOI: 10.1107/S1600536811015017/is2689sup1.cif

Structure factors: contains datablocks I. DOI: 10.1107/S1600536811015017/is2689Isup2.hkl

Supplementary material file. DOI: 10.1107/S1600536811015017/is2689Isup3.cml

Additional supplementary materials:  crystallographic information; 3D view; checkCIF report

**Fig. 1.**
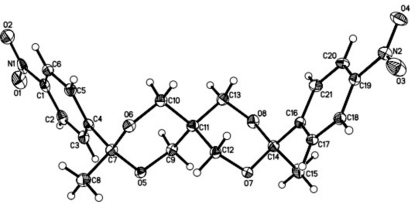
The molecular structure of the title compound. Displacement ellipsoids are drawn at the 30% probability level.

Enhanced figure: interactive version of Fig. 1

## Figures and Tables

**Table 1:** Hydrogen-bond geometry (Å, °)

*D*—H⋯*A*	*D*—H	H⋯*A*	*D*⋯*A*	*D*—H⋯*A*
C9—H9*A*⋯O2^i^	0.97	2.56	3.515 (3)	168
C10—H10*B*⋯O1^i^	0.97	2.59	3.533 (3)	164
C17—H17⋯O4^ii^	0.93	2.45	3.337 (3)	160
C20—H20⋯O7^iii^	0.93	2.37	3.242 (3)	155

Symmetry codes: (i) 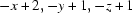; (ii) 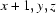; (iii) 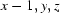.

## supplementary crystallographic information

### Comment

Owing to the characteristic axial and helical chirality, the stereochemistry of spiranes with six-membered rings has been extensively studied (Cismaş *et al*., 2005). In the past three decades, most of these investigations were carried out with spiranes containing 1,3-dioxane units (Mihiş *et al.*, 2008; Sun *et al.*, 2010). We herein present the structure of 3,9-dimethyl-3,9-di(4-nitrophenyl)-2,4,8,10-tetraoxaspiro[5.5]undecane (Fig. 1).

In the title compound, the two non-planar six-membered heterocycle adopt chair conformations. The dihedral angle between the nitrobenzene rings is 58.22 (11)°. In the crystal structure, weak intermolecular C—H···O interactions contribute to the crystal packing (Table 1).

### Experimental

To a solution of *p*-nitroacetophen (2.06 g, 12.5 mmol) and pentaerythritol (0.68 g, 5 mmol) in toluene(30 ml), *p*-toluenesulfonic acid (0.05 g, 0.3 mmol) as catalyst was added, respectively. Then, the mixtures were refluxed for 6 h to complete the reaction. After reaction, the mixtures were allowed to cool to the room temperature, chloroform (30 ml) was added to dissolve the product, the remain residue was purified by recrystallization using ethanol to provide the title compound as a white solid (85% yield, m.p. 516–517 K). Single crystals suitable for X-ray diffraction were obtained by evaporation of an ethanol and chloroform mixed solution.

### Refinement

12 restraints with the ISOR command was applied to make O2 and N1 be approximately isotropic. All the H atoms were placed in geometrically idealized positions and constrained to ride on their parent atoms, with C—H distances of 0.93–0.97 Å, and with *U*_iso_(H) = 1.2*U*_eq_(C) or 1.5*U*_eq_(methyl C).

### Crystal data

**Table:**

C_21_H_22_N_2_O_8_	*Z* = 2
*M**_r_* = 430.41	*F*(000) = 452
Triclinic, *P*1	*D*_x_ = 1.392 Mg m^−^^3^
Hall symbol: -P 1	Mo *K*α radiation, λ = 0.71073 Å
*a* = 7.4215 (12) Å	Cell parameters from 3502 reflections
*b* = 11.8790 (18) Å	θ = 2.9–30.2°
*c* = 13.522 (3) Å	µ = 0.11 mm^−^^1^
α = 115.280 (4)°	*T* = 295 K
β = 94.426 (4)°	Block, colorless
γ = 103.444 (3)°	0.21 × 0.21 × 0.16 mm
*V* = 1027.0 (3) Å^3^	

### Data collection

**Table:**

Bruker APEXII CCD diffractometer	3563 independent reflections
Radiation source: fine-focus sealed tube	2980 reflections with *I* > 2σ(*I*)
graphite	*R*_int_ = 0.022
φ and ω scans	θ_max_ = 25.0°, θ_min_ = 1.7°
Absorption correction: multi-scan (*SADABS*; Sheldrick, 2003)	*h* = −8→8
*T*_min_ = 0.976, *T*_max_ = 0.986	*k* = −14→14
5588 measured reflections	*l* = −16→11

### Refinement

**Table:**

Refinement on *F*^2^	Primary atom site location: structure-invariant direct methods
Least-squares matrix: full	Secondary atom site location: difference Fourier map
*R*[*F*^2^ > 2σ(*F*^2^)] = 0.051	Hydrogen site location: inferred from neighbouring sites
*wR*(*F*^2^) = 0.187	H-atom parameters constrained
*S* = 1.07	*w* = 1/[σ^2^(*F*_o_^2^) + (0.135*P*)^2^ + 0.1621*P*] where *P* = (*F*_o_^2^ + 2*F*_c_^2^)/3
3563 reflections	(Δ/σ)_max_ < 0.001
282 parameters	Δρ_max_ = 0.37 e Å^−^^3^
12 restraints	Δρ_min_ = −0.26 e Å^−^^3^

### Special details

**Table:**

Geometry. All e.s.d.'s (except the e.s.d. in the dihedral angle between two l.s. planes) are estimated using the full covariance matrix. The cell e.s.d.'s are taken into account individually in the estimation of e.s.d.'s in distances, angles and torsion angles; correlations between e.s.d.'s in cell parameters are only used when they are defined by crystal symmetry. An approximate (isotropic) treatment of cell e.s.d.'s is used for estimating e.s.d.'s involving l.s. planes.
Refinement. Refinement of *F*^2^ against ALL reflections. The weighted *R*-factor *wR* and goodness of fit *S* are based on *F*^2^, conventional *R*-factors *R* are based on *F*, with *F* set to zero for negative *F*^2^. The threshold expression of *F*^2^ > σ(*F*^2^) is used only for calculating *R*-factors(gt) *etc*. and is not relevant to the choice of reflections for refinement. *R*-factors based on *F*^2^ are statistically about twice as large as those based on *F*, and *R*- factors based on ALL data will be even larger.

### Fractional atomic coordinates and isotropic or equivalent isotropic displacement parameters (Å^2^)

**Table:**

	*x*	*y*	*z*	*U*_iso_*/*U*_eq_	
N1	1.2247 (3)	0.6344 (2)	0.63022 (16)	0.0321 (5)	
N2	0.1620 (3)	0.14798 (18)	−0.42929 (16)	0.0254 (5)	
O1	1.2782 (3)	0.5766 (2)	0.67765 (15)	0.0451 (5)	
O2	1.1755 (3)	0.73336 (19)	0.67867 (14)	0.0424 (5)	
O3	0.2195 (3)	0.18113 (19)	−0.49758 (15)	0.0398 (5)	
O4	−0.0012 (2)	0.13861 (17)	−0.41184 (15)	0.0346 (4)	
O5	1.2135 (2)	0.31399 (14)	0.10612 (12)	0.0205 (4)	
O6	1.1156 (2)	0.48954 (14)	0.11022 (12)	0.0209 (4)	
O7	0.86957 (19)	0.10427 (13)	−0.16096 (12)	0.0192 (4)	
O8	0.6406 (2)	0.06651 (14)	−0.06059 (12)	0.0238 (4)	
C1	1.2214 (3)	0.5840 (2)	0.51053 (18)	0.0242 (5)	
C2	1.2715 (3)	0.4717 (2)	0.45462 (19)	0.0254 (5)	
H2A	1.3038	0.4270	0.4920	0.030*	
C3	1.2728 (3)	0.4269 (2)	0.34267 (18)	0.0231 (5)	
H3A	1.3069	0.3515	0.3041	0.028*	
C4	1.2233 (3)	0.4939 (2)	0.28631 (17)	0.0195 (5)	
C5	1.1707 (3)	0.6059 (2)	0.34539 (18)	0.0224 (5)	
H5	1.1353	0.6501	0.3083	0.027*	
C6	1.1703 (3)	0.6524 (2)	0.45800 (18)	0.0244 (5)	
H6	1.1366	0.7278	0.4973	0.029*	
C7	1.2414 (3)	0.4498 (2)	0.16478 (17)	0.0199 (5)	
C8	1.4377 (3)	0.5140 (2)	0.15867 (19)	0.0264 (5)	
H8A	1.5284	0.4917	0.1958	0.040*	
H8B	1.4615	0.6069	0.1944	0.040*	
H8C	1.4484	0.4846	0.0819	0.040*	
C9	1.0213 (3)	0.2363 (2)	0.08616 (17)	0.0215 (5)	
H9A	0.9863	0.2480	0.1568	0.026*	
H9B	1.0111	0.1449	0.0421	0.026*	
C10	0.9207 (3)	0.4212 (2)	0.09267 (17)	0.0211 (5)	
H10A	0.8431	0.4507	0.0534	0.025*	
H10B	0.8844	0.4397	0.1641	0.025*	
C11	0.8872 (3)	0.2752 (2)	0.02479 (17)	0.0200 (5)	
C12	0.9199 (3)	0.2421 (2)	−0.09323 (17)	0.0187 (5)	
H12A	0.8447	0.2786	−0.1264	0.022*	
H12B	1.0520	0.2806	−0.0901	0.022*	
C13	0.6814 (3)	0.2026 (2)	0.01311 (18)	0.0236 (5)	
H13A	0.6576	0.2144	0.0859	0.028*	
H13B	0.5984	0.2386	−0.0156	0.028*	
C14	0.6799 (3)	0.0390 (2)	−0.16773 (17)	0.0205 (5)	
C15	0.6618 (3)	−0.1047 (2)	−0.22524 (19)	0.0282 (5)	
H15A	0.7513	−0.1229	−0.1834	0.042*	
H15B	0.6865	−0.1289	−0.2991	0.042*	
H15C	0.5360	−0.1537	−0.2298	0.042*	
C16	0.5415 (3)	0.07090 (19)	−0.23489 (17)	0.0192 (5)	
C17	0.5956 (3)	0.0991 (2)	−0.31997 (17)	0.0195 (5)	
H17	0.7169	0.1014	−0.3338	0.023*	
C18	0.4722 (3)	0.1238 (2)	−0.38398 (17)	0.0204 (5)	
H18	0.5097	0.1443	−0.4398	0.024*	
C19	0.2916 (3)	0.1176 (2)	−0.36354 (17)	0.0208 (5)	
C20	0.2302 (3)	0.0863 (2)	−0.28153 (18)	0.0232 (5)	
H20	0.1073	0.0812	−0.2699	0.028*	
C21	0.3573 (3)	0.0631 (2)	−0.21751 (18)	0.0236 (5)	
H21	0.3192	0.0419	−0.1621	0.028*	

### Atomic displacement parameters (Å^2^)

**Table:**

	*U*^11^	*U*^22^	*U*^33^	*U*^12^	*U*^13^	*U*^23^
N1	0.0187 (10)	0.0450 (13)	0.0191 (10)	−0.0053 (9)	−0.0021 (8)	0.0112 (9)
N2	0.0205 (10)	0.0278 (10)	0.0265 (10)	0.0095 (8)	0.0042 (8)	0.0100 (9)
O1	0.0418 (11)	0.0681 (14)	0.0253 (9)	0.0071 (10)	0.0027 (8)	0.0266 (10)
O2	0.0362 (10)	0.0535 (12)	0.0220 (9)	0.0097 (9)	0.0090 (8)	0.0045 (8)
O3	0.0348 (10)	0.0605 (13)	0.0410 (11)	0.0205 (9)	0.0092 (8)	0.0349 (10)
O4	0.0220 (9)	0.0451 (10)	0.0416 (10)	0.0175 (8)	0.0069 (7)	0.0203 (9)
O5	0.0209 (8)	0.0203 (8)	0.0193 (8)	0.0082 (6)	0.0035 (6)	0.0072 (6)
O6	0.0240 (8)	0.0224 (8)	0.0189 (8)	0.0084 (6)	0.0036 (6)	0.0111 (6)
O7	0.0163 (7)	0.0208 (8)	0.0186 (7)	0.0058 (6)	0.0036 (6)	0.0072 (6)
O8	0.0253 (8)	0.0256 (8)	0.0206 (8)	0.0037 (7)	0.0038 (6)	0.0127 (7)
C1	0.0158 (10)	0.0328 (12)	0.0165 (11)	−0.0022 (9)	0.0014 (8)	0.0097 (10)
C2	0.0214 (11)	0.0306 (12)	0.0237 (11)	0.0004 (9)	−0.0023 (9)	0.0168 (10)
C3	0.0224 (11)	0.0208 (11)	0.0239 (11)	0.0031 (9)	0.0002 (9)	0.0106 (9)
C4	0.0149 (10)	0.0222 (11)	0.0194 (11)	0.0027 (8)	0.0020 (8)	0.0094 (9)
C5	0.0220 (11)	0.0233 (11)	0.0216 (11)	0.0064 (9)	0.0024 (9)	0.0105 (9)
C6	0.0191 (11)	0.0256 (11)	0.0216 (11)	0.0041 (9)	0.0042 (9)	0.0059 (9)
C7	0.0225 (11)	0.0203 (11)	0.0185 (11)	0.0082 (9)	0.0037 (9)	0.0096 (9)
C8	0.0265 (12)	0.0297 (12)	0.0236 (12)	0.0069 (10)	0.0086 (9)	0.0131 (10)
C9	0.0250 (11)	0.0197 (11)	0.0180 (10)	0.0056 (9)	0.0031 (9)	0.0076 (9)
C10	0.0231 (11)	0.0253 (11)	0.0159 (10)	0.0092 (9)	0.0032 (8)	0.0095 (9)
C11	0.0217 (11)	0.0234 (11)	0.0174 (11)	0.0086 (9)	0.0057 (8)	0.0103 (9)
C12	0.0188 (10)	0.0192 (10)	0.0175 (11)	0.0048 (8)	0.0030 (8)	0.0085 (9)
C13	0.0245 (11)	0.0287 (12)	0.0171 (10)	0.0089 (9)	0.0056 (9)	0.0092 (9)
C14	0.0191 (11)	0.0227 (11)	0.0184 (11)	0.0054 (9)	0.0040 (8)	0.0086 (9)
C15	0.0284 (12)	0.0228 (12)	0.0296 (12)	0.0057 (10)	−0.0020 (10)	0.0107 (10)
C16	0.0179 (10)	0.0158 (10)	0.0202 (11)	0.0043 (8)	0.0030 (8)	0.0055 (9)
C17	0.0163 (10)	0.0228 (11)	0.0174 (10)	0.0056 (8)	0.0059 (8)	0.0070 (9)
C18	0.0188 (10)	0.0234 (11)	0.0171 (10)	0.0045 (9)	0.0057 (8)	0.0080 (9)
C19	0.0193 (11)	0.0186 (10)	0.0199 (11)	0.0065 (8)	0.0006 (8)	0.0048 (9)
C20	0.0167 (10)	0.0281 (12)	0.0242 (11)	0.0080 (9)	0.0090 (9)	0.0099 (10)
C21	0.0206 (11)	0.0268 (12)	0.0243 (11)	0.0054 (9)	0.0087 (9)	0.0127 (10)

### Geometric parameters (Å, °)

**Table:**

N1—O1	1.226 (3)	C8—H8C	0.9600
N1—O2	1.234 (3)	C9—C11	1.519 (3)
N1—C1	1.462 (3)	C9—H9A	0.9700
N2—O3	1.218 (2)	C9—H9B	0.9700
N2—O4	1.240 (2)	C10—C11	1.522 (3)
N2—C19	1.466 (3)	C10—H10A	0.9700
O5—C7	1.414 (2)	C10—H10B	0.9700
O5—C9	1.441 (2)	C11—C12	1.526 (3)
O6—C7	1.426 (2)	C11—C13	1.528 (3)
O6—C10	1.430 (2)	C12—H12A	0.9700
O7—C14	1.415 (2)	C12—H12B	0.9700
O7—C12	1.428 (2)	C13—H13A	0.9700
O8—C14	1.413 (2)	C13—H13B	0.9700
O8—C13	1.431 (3)	C14—C15	1.510 (3)
C1—C2	1.379 (3)	C14—C16	1.534 (3)
C1—C6	1.379 (3)	C15—H15A	0.9600
C2—C3	1.375 (3)	C15—H15B	0.9600
C2—H2A	0.9300	C15—H15C	0.9600
C3—C4	1.400 (3)	C16—C17	1.391 (3)
C3—H3A	0.9300	C16—C21	1.393 (3)
C4—C5	1.393 (3)	C17—C18	1.376 (3)
C4—C7	1.525 (3)	C17—H17	0.9300
C5—C6	1.381 (3)	C18—C19	1.381 (3)
C5—H5	0.9300	C18—H18	0.9300
C6—H6	0.9300	C19—C20	1.388 (3)
C7—C8	1.506 (3)	C20—C21	1.384 (3)
C8—H8A	0.9600	C20—H20	0.9300
C8—H8B	0.9600	C21—H21	0.9300
			
O1—N1—O2	123.3 (2)	C11—C10—H10B	109.6
O1—N1—C1	118.2 (2)	H10A—C10—H10B	108.1
O2—N1—C1	118.6 (2)	C9—C11—C10	107.29 (16)
O3—N2—O4	123.52 (19)	C9—C11—C12	111.57 (17)
O3—N2—C19	118.69 (18)	C10—C11—C12	110.46 (16)
O4—N2—C19	117.79 (18)	C9—C11—C13	111.03 (16)
C7—O5—C9	113.97 (15)	C10—C11—C13	109.82 (17)
C7—O6—C10	113.62 (14)	C12—C11—C13	106.70 (17)
C14—O7—C12	113.49 (15)	O7—C12—C11	110.82 (16)
C14—O8—C13	113.91 (15)	O7—C12—H12A	109.5
C2—C1—C6	122.4 (2)	C11—C12—H12A	109.5
C2—C1—N1	119.3 (2)	O7—C12—H12B	109.5
C6—C1—N1	118.3 (2)	C11—C12—H12B	109.5
C3—C2—C1	118.90 (19)	H12A—C12—H12B	108.1
C3—C2—H2A	120.5	O8—C13—C11	110.95 (16)
C1—C2—H2A	120.5	O8—C13—H13A	109.4
C2—C3—C4	120.5 (2)	C11—C13—H13A	109.4
C2—C3—H3A	119.7	O8—C13—H13B	109.4
C4—C3—H3A	119.7	C11—C13—H13B	109.4
C5—C4—C3	118.81 (19)	H13A—C13—H13B	108.0
C5—C4—C7	121.08 (18)	O8—C14—O7	111.31 (16)
C3—C4—C7	119.96 (19)	O8—C14—C15	106.00 (17)
C6—C5—C4	121.19 (19)	O7—C14—C15	106.04 (16)
C6—C5—H5	119.4	O8—C14—C16	111.73 (16)
C4—C5—H5	119.4	O7—C14—C16	111.13 (16)
C1—C6—C5	118.1 (2)	C15—C14—C16	110.34 (17)
C1—C6—H6	120.9	C14—C15—H15A	109.5
C5—C6—H6	120.9	C14—C15—H15B	109.5
O5—C7—O6	111.16 (15)	H15A—C15—H15B	109.5
O5—C7—C8	106.19 (17)	C14—C15—H15C	109.5
O6—C7—C8	106.18 (16)	H15A—C15—H15C	109.5
O5—C7—C4	112.31 (16)	H15B—C15—H15C	109.5
O6—C7—C4	110.65 (16)	C17—C16—C21	119.12 (19)
C8—C7—C4	110.07 (17)	C17—C16—C14	119.69 (18)
C7—C8—H8A	109.5	C21—C16—C14	121.04 (18)
C7—C8—H8B	109.5	C18—C17—C16	120.94 (19)
H8A—C8—H8B	109.5	C18—C17—H17	119.5
C7—C8—H8C	109.5	C16—C17—H17	119.5
H8A—C8—H8C	109.5	C17—C18—C19	118.60 (19)
H8B—C8—H8C	109.5	C17—C18—H18	120.7
O5—C9—C11	110.63 (16)	C19—C18—H18	120.7
O5—C9—H9A	109.5	C18—C19—C20	122.33 (19)
C11—C9—H9A	109.5	C18—C19—N2	118.64 (18)
O5—C9—H9B	109.5	C20—C19—N2	119.01 (18)
C11—C9—H9B	109.5	C21—C20—C19	118.04 (19)
H9A—C9—H9B	108.1	C21—C20—H20	121.0
O6—C10—C11	110.27 (16)	C19—C20—H20	121.0
O6—C10—H10A	109.6	C20—C21—C16	120.92 (19)
C11—C10—H10A	109.6	C20—C21—H21	119.5
O6—C10—H10B	109.6	C16—C21—H21	119.5
			
O1—N1—C1—C2	2.3 (3)	C14—O7—C12—C11	57.4 (2)
O2—N1—C1—C2	−178.41 (19)	C9—C11—C12—O7	66.9 (2)
O1—N1—C1—C6	−176.8 (2)	C10—C11—C12—O7	−173.87 (15)
O2—N1—C1—C6	2.5 (3)	C13—C11—C12—O7	−54.5 (2)
C6—C1—C2—C3	0.8 (3)	C14—O8—C13—C11	−56.1 (2)
N1—C1—C2—C3	−178.31 (18)	C9—C11—C13—O8	−68.0 (2)
C1—C2—C3—C4	−0.3 (3)	C10—C11—C13—O8	173.56 (15)
C2—C3—C4—C5	−0.7 (3)	C12—C11—C13—O8	53.8 (2)
C2—C3—C4—C7	174.82 (19)	C13—O8—C14—O7	55.3 (2)
C3—C4—C5—C6	1.2 (3)	C13—O8—C14—C15	170.19 (16)
C7—C4—C5—C6	−174.24 (19)	C13—O8—C14—C16	−69.6 (2)
C2—C1—C6—C5	−0.3 (3)	C12—O7—C14—O8	−55.9 (2)
N1—C1—C6—C5	178.84 (18)	C12—O7—C14—C15	−170.77 (15)
C4—C5—C6—C1	−0.8 (3)	C12—O7—C14—C16	69.3 (2)
C9—O5—C7—O6	−54.6 (2)	O8—C14—C16—C17	155.17 (18)
C9—O5—C7—C8	−169.62 (16)	O7—C14—C16—C17	30.2 (2)
C9—O5—C7—C4	70.0 (2)	C15—C14—C16—C17	−87.2 (2)
C10—O6—C7—O5	55.6 (2)	O8—C14—C16—C21	−29.4 (3)
C10—O6—C7—C8	170.66 (16)	O7—C14—C16—C21	−154.43 (19)
C10—O6—C7—C4	−69.9 (2)	C15—C14—C16—C21	88.2 (2)
C5—C4—C7—O5	−154.14 (18)	C21—C16—C17—C18	2.3 (3)
C3—C4—C7—O5	30.5 (3)	C14—C16—C17—C18	177.83 (18)
C5—C4—C7—O6	−29.3 (3)	C16—C17—C18—C19	−1.2 (3)
C3—C4—C7—O6	155.32 (18)	C17—C18—C19—C20	−0.5 (3)
C5—C4—C7—C8	87.8 (2)	C17—C18—C19—N2	178.06 (18)
C3—C4—C7—C8	−87.6 (2)	O3—N2—C19—C18	−2.0 (3)
C7—O5—C9—C11	55.9 (2)	O4—N2—C19—C18	178.18 (19)
C7—O6—C10—C11	−57.5 (2)	O3—N2—C19—C20	176.7 (2)
O5—C9—C11—C10	−54.6 (2)	O4—N2—C19—C20	−3.2 (3)
O5—C9—C11—C12	66.6 (2)	C18—C19—C20—C21	1.1 (3)
O5—C9—C11—C13	−174.56 (16)	N2—C19—C20—C21	−177.47 (18)
O6—C10—C11—C9	55.5 (2)	C19—C20—C21—C16	0.0 (3)
O6—C10—C11—C12	−66.3 (2)	C17—C16—C21—C20	−1.7 (3)
O6—C10—C11—C13	176.29 (15)	C14—C16—C21—C20	−177.16 (19)

### Hydrogen-bond geometry (Å, °)

**Table:**

*D*—H···*A*	*D*—H	H···*A*	*D*···*A*	*D*—H···*A*
C9—H9A···O2^i^	0.97	2.56	3.515 (3)	168.
C10—H10B···O1^i^	0.97	2.59	3.533 (3)	164.
C17—H17···O4^ii^	0.93	2.45	3.337 (3)	160.
C20—H20···O7^iii^	0.93	2.37	3.242 (3)	155.

Symmetry codes: (i) −*x*+2, −*y*+1, −*z*+1; (ii) *x*+1, *y*, *z*; (iii) *x*−1, *y*, *z*.
